# Management for Cervical Cancer Patients: A Comparison of the Guidelines from the International Scientific Societies (ESGO-NCCN-ASCO-AIOM-FIGO-BGCS-SEOM-ESMO-JSGO)

**DOI:** 10.3390/cancers16142541

**Published:** 2024-07-15

**Authors:** Stefano Restaino, Giulia Pellecchia, Martina Arcieri, Giorgio Bogani, Cristina Taliento, Pantaleo Greco, Lorenza Driul, Vito Chiantera, Alfredo Ercoli, Francesco Fanfani, Anna Fagotti, Andrea Ciavattini, Giovanni Scambia, Giuseppe Vizzielli, Gynecologic Oncology Group

**Affiliations:** 1 Clinic of Obstetrics and Gynecology, “Santa Maria della Misericordia” University Hospital, Azienda Sanitaria Universitaria Friuli Centrale, 33100 Udine, Italy; stefano.restaino@asufc.sanita.fvg.it (S.R.); pellecchia.giulia001@spes.uniud.it (G.P.); lorenza.driul@uniud.it (L.D.); giuseppe.vizzielli@uniud.it (G.V.); 2 PhD School in Biomedical Sciences, Gender Medicine, Child and Women Health, University of Sassari, 07100 Sassari, Italy; 3 Department of Medicine, University of Udine, 33100 Udine, Italy; 4 Gynaecological Oncology Unit, Fondazione IRCCS Istituto Nazionale dei Tumori di Milano, 20133 Milano, Italy; giorgio.bogani@istitutotumori.mi.it; 5 Department of Medical Sciences, Obstetrics and Gynecology Unit, University of Ferrara, 44121 Ferrara, Italy; cristina.taliento@unife.it (C.T.); pantaleo.greco@unife.it (P.G.); 6 Department of Development and Regeneration, Woman and Child, KU Leuven, 3000 Leuven, Belgium; 7 Department of Health Promotion, Mother and Child Care, Internal Medicine and Medical Specialties (PROMISE), University of Palermo, 90133 Palermo, Italy; vito.chiantera@istitutotumori.na.it; 8 Unit of Gynecologic Oncology, National Cancer Institute, IRCCS, Fondazione “G. Pascale”, 80131 Naples, Italy; 9 Department of Human Pathology in Adult and Childhood “G. Barresi”, University of Messina, 98122 Messina, Italy; alfredo.ercoli@unime.it; 10 Gynecologic Oncology Unit, Department of Woman and Child Health and Public Health, Fondazione Policlinico Universitario Agostino Gemelli IRCCS, 00168 Rome, Italy; francesco.fanfani@policlinicogemelli.it (F.F.); anna.fagotti@policlinicogemelli.it (A.F.); giovanni.scambia@unicatt.it (G.S.); 11 Woman’s Health Sciences Department, Gynecologic Section, Polytechnic University of Marche, 60121 Ancona, Italy; a.ciavattini@staff.univpm.it

**Keywords:** cervical cancer, management, guidelines, narrative review

## Abstract

**Simple Summary:**

Cervical cancer, a very aggressive gynecological malignancy that also affects young women, remains significantly prevalent despite worldwide efforts in HPV vaccination campaigns. Cervical cancer research is experiencing a period of significant change, with intense ongoing debates on issues that could potentially transform current guidelines. Therefore, in light of these changes, guidelines and protocols will soon need significant updates. Hence, this paper aims to summarize and compare the most recent recommendations published by international gynecological oncology societies for patients with cervical cancer. A comparative analysis of American, Asian, and European guidelines was conducted to evaluate the different recommendations for diagnostic, surgical, medical, and follow-up management.

**Abstract:**

Cervical cancer continues to have a significant incidence, despite global efforts in HPV vaccination campaigns. Managing this condition involves a diverse team of healthcare professionals. Research in this field is undergoing a period of great revolution in multiple areas, and international guidelines will soon have to adapt to new scientific evidence. This could be true mainly in locally advanced stages, and it could also be true for minimal invasive surgery. This paper aims to summarize and compare the most recent recommendations published by international gynecological oncological societies for patients with cervical cancer. From their comparison, common aspects and disagreements emerged, especially in the diagnostic pathway and follow-up strategies. Several issues that remain to be debated in the literature were addressed and compared, highlighting similarities and differences, from the role of the sentinel lymph node in early stages to that of the adjuvant hysterectomy in locally advanced tumors. On the surgical side, for this last subset of patients, currently, a laparotomic approach is recommended. At the same time, the advent of immunotherapy has just opened up new and promising scenarios in systemic treatment for locally advanced cervical cancer, and international guidelines will soon introduce it into their algorithms.

## 1. Introduction

Cervical cancer ranks as the fourth most frequent cancer in women, and affects mainly younger age groups. Despite the effectiveness of HPV screening and vaccination initiatives, the mortality rate due to delayed diagnosis has significantly increased in regions with developing economies, where screening initiatives lack proper organization [1]. Here, an estimated 84% of cervical cancer cases still occur [1]. Worldwide, 604,000 new cases of cervical cancer and 342,000 deaths were recorded in 2020 [2]. In developed countries, screening and prevention programs are systematically organized and planned, such as those recommended by FIGO to reduce the incidence and mortality associated with cervical cancer [3]. In recent decades, we are assisting rapid changes in the management of these patients. From the surgical point of view, in 2018, Ramirez et al. were said to have ‘come back from the future’ with the LACC trial, while the advent of immunotherapy has changed the medical approach to this pathology [4]. The LACC trial, a multicenter randomized clinical trial, has revealed substantial adverse effects on survival outcomes in early-stage cervical cancer patients undergoing minimally invasive surgery (MIS) compared to the laparotomy [5]. Since that time, there has been a quick evolution and continuous updating in the surgical domain regarding the management of patients with cervical cancer. Numerous scientific papers have been published after that, either for or against this hypothesis, but the scientific debate remains substantially open. Lastly, the recent release of the SHAPE trial results has shown that simple hysterectomy does not exhibit inferiority over radical hysterectomy in terms of survival and recurrence for the surgical treatment of early-stage cervical carcinomas [6]. On the other hand, immunotherapy for the treatment of locally advanced or metastatic cervical cancer has demonstrated substantially prolonged progression-free and overall survival in comparison to a placebo [7,8]. Similar issues have also been observed in patients experiencing persistent or recurrent disease [9,10]. This will likely prompt a significant demand for the revision of clinical recommendations in the care of patients with cervical cancer. This progress is demonstrated by the publication and update of the several guidelines. The most recent in this field are the European ESGO/ESTRO/ESP guidelines [11] and the American ones (NCCN) [12]. In light of the changes introduced by the LACC and SHAPE trial, followed by undeniable innovations in medical treatment, swift revisions to national and international guidelines became imperative [13]. Consequently, there is a notable interest in scrutinizing and comparing the diverse guidelines in response to these updates. This paper aims just to summarize the more recently published guidelines from leading scientific gynecology oncological societies to provide quickly accessible data for healthcare professionals involved in this field.

## 2. Materials and Methods

This is a descriptive comparative review. We searched PubMed and the websites of all international scientific societies of gynecological oncology for the most recent guidelines published in Africa, America, Asia, Australia, and Europe. Inclusion criteria were as follows: papers produced in the least years on the topic of cervical cancer topic in the English language. Exclusion criteria were: non-English language drafting and one version prior to the latest from each scientific society.

Poland’s medical guidelines align with those of ASCO, but there is a lower rate of women undergoing chemotherapy in this country when compared to others with lower survival rates for the disease [14]. No African recommendations were identified. In total, 13 scientific recommendations were finally included in the revision. A brief summary of them can be found in Table 1.

For the pathological staging of cervical cancer in patients undergoing surgical procedures, there are two classifications, as follows: the 2018 clinical and radiological FIGO (International Federation of Gynecology and Obstetrics) staging, and the 2010 pathological AJCC (American Joint Committee on Cancer) [18,24]. That being said, the 2018 FIGO staging is commonly recognized in all scientific recommendations included for the clinical and radiological preoperative tumor assessment. However, not all guidelines refer to the same staging when stating their recommendations. ASCO, ESMO, AIOM, SEOM, BCGS, and JSGO state their recommendations, relying on FIGO staging. Otherwise, the authors of the ESGO guidelines, the main European society in Gynecological oncology, refer to the AJCCs. American NCCN refers to both, predominantly FIGO. Henceforth, since they are overlapping and corresponding, the two pathological stages will be referenced according to the remarks provided in each respective guideline [18].

## 3. Results

Similarities and differences that emerged from the comparison of these guidelines are systematically reported below. In particular, the adopted method followed the diagnostic and therapeutic work-up that cervical cancer’s patients undergo in clinical practice.

### 3.1. Diagnosis of Cervical Neoplasm

Early-stage cervical cancer typically presents without symptoms, emphasizing the importance of screening for diagnosis. In symptomatic patients, the most common manifestations include irregular or heavy vaginal bleeding and/or postcoital bleeding [25]. The diagnosis of cervical neoplasia is histologic. Diagnostic suspicion in initial cases is based on a doubtful or positive Pap test report (level I examination), followed by colposcopy (level II examination), which allows for targeted biopsy and histological sampling. In some cases, conization may be necessary if biopsy is inadequate to define invasiveness. Bimanual gynecological examination must be conducted in all patients with a confirmed or suspected diagnosis of portio neoplasm. Up to this point, these diagnostic steps are encompassed in the various papers under review. The NCCN guidelines recommended, for younger patients, testing for HIV after providing appropriate counseling regarding lifestyle choices [12]. If there are indications of a locally advanced lesion based on objective gynecological assessment and/or instrumental examinations, a gynecological examination under narcosis (EUA) may be conducted. This would involve a thorough inspection of the vagina and cervix, along with a combined vaginal and rectal examination to meticulously evaluate the extent of the disease. Any suspected cervical and/or vaginal lesions should be biopsied. If bladder and/or rectal infiltration is suspected, a cystoscopy and/or rectoscopy with possible mucosal biopsies should also be performed. ESGO and NCCN guidelines emphasize the potential need for cystoscopy and/or rectoscopy in cases where there is a suspicion of disease extension [11,12]. Regarding EUA, there is no specific mention in the American and European ESGO guidelines [11,12]. The AIOM and British guidelines consider the EUA to be an available tool to be performed if necessary or at the discretion of the individual operator [17,19]. In the Spanish guidelines, it is mentioned as an alternative to an unsatisfactory pelvic examination, and the ESMO authors suggest this evaluation in suspicion of vaginal/parametrial involvement performed by a gynecological oncologist and a radiation oncologist [20,21]. The evaluation of tumor marker squamous cell carcinoma antigens (SCC-TA4) is still absent in all international guidelines, except in the Italian AIOM ones, due to the need for further validation [26].

### 3.2. Imaging

Imaging plays an important role in the diagnosis of cervical cancer. Over the past two decades, ultrasound has gained interest in the study of the cervix, demonstrating high levels of accuracy even for small tumors. Its advantage is that it offers a dynamic examination that allows the operator to assess how adjacent tissues slide against each other. This is crucial in determining the local extent of tumors [27]. Despite this, it is still not universally recognized as a diagnostic imaging method by international guidelines. The ESGO/ESTRO/ESP 2023 guidelines and FIGO report place it as a valid diagnostic option, but only if performed by experienced sonographers [11,28]. The NCCN2024 and British guidelines consider it to be an alternative to magnetic resonance imaging (MRI) [12,19]. In contrast, the JSGO, ESMO, AIOM, and SEOM guidelines do not mention it in the diagnostic workup [15,17,20,21]. MRI is the first-choice imaging for the evaluation of local tumor extent recommended by the European Society of Urogenital Radiology (ESUR) [25], and it is unanimously recognized by all international gynecologic oncology societies in the staging of cervical cancer [17]. 2-deoxy-2-18 F Fluro-D-glucose positron-emission tomography (18F-FDG PET/CT) is recommended to assess the extent of loco-regional, lymph node, and metastatic disease. In cases of locally advanced tumors, total body computed tomography (CT) or chest CT with contrast may be an alternative to PET-CT for studying distant disease, particularly when PET-CT is not available. Both are valuable diagnostic methods for assessing lymph node involvement or distant disease. However, their role in assessing the primary tumor is considered to be secondary. The enhanced sensitivity of PET/CT compared to CT alone is particularly advantageous for detecting abdominal lymph node metastases. Table 2 compares the diagnostic workup of patients with cervical cancer. In particular, we could notice that EUA is not routinely recommended in the work-up, and there is significant variability in the choice of imaging among scientific societies. ASCOs and JSGO do not provide information regarding the diagnostic work-up.

### 3.3. Early-Stage Cervical Cancer Surgical Staging

The most recent and robust scientific evidence supports the treatment of patients with early-stage disease with a surgical approach. Standard surgery involves radical hysterectomy combined with systematic pelvic lymphadenectomy. In stage IA1, in the absence of invasion of lymphovascular spaces, both conization and extrafascial hysterectomy are possible without lymph node dissection. Table 3 schematically lists the therapeutic indications inherent in each guideline document examined for immediate comparison. The ESGO and NCCN guidelines distinguish stage IA2 based on the presence or absence of lymphovascular space invasion (LVSI). However, it should be noted that in stages IA2, NCCN places an unequivocal indication for radical hysterectomy with lymph node staging [12]. In cases with negative LVSI, the ESGO recommendation is for simple conization or extrafascial hysterectomy, with the possibility of sentinel node biopsy. Regarding LVSI positivity, both guidelines suggest sentinel biopsy [9]. Overall, for stages T1b1, T1b2, and T2a1, ESGO discriminates treatment based on the radiological assessment of lymph node status [9]. Upon confirming lymph node negativity, the approach involves radical hysterectomy (the degree of radicality is determined based on the preoperative risk category), pelvic lymphadenectomy, and bilateral adnexectomy. For women of reproductive age diagnosed with squamous cell carcinoma, the possibility of ovarian preservation should be discussed. It may be considered in cases of HPV-associated squamous carcinoma or adenocarcinoma, but it is not recommended for HPV-independent adenocarcinomas. If ovaries are preserved, opportunistic bilateral salpingectomy should be performed [11]. Alternatively, exclusive radio-chemotherapy remains viable if known risk factors at diagnosis could predict adjuvant radiotherapy [11]. Conversely, if lymph node status is positive on imaging, the therapeutic choice is exclusive radio-chemotherapy. Prognostic factors correlated with increased risk of lymph node metastasis even in early stages and reduced disease-free survival (DFS) were as follows: tumor size, the presence or absence of LVSI, and the extent of stromal invasion. Assessing LVSI through a semi-quantitative approach (distinguishing into “negative”, “focal positive”, and “diffuse positive”), seems to be beneficial in identifying high-risk patients who are likely to experience shorter DFS, as well as an increased likelihood of lymphatic and distant recurrences, particularly in individuals with early-stage disease [29]. The combination of these three factors has resulted in a stratification into risk classes (low, intermediate, high). The AIOM guidelines go a step further by drawing up a table combining the risk class with the various prognostic factors and the type of radical hysterectomy according to the Querleu–Morrow classification [30]. In the SEOM guideline, the sentinel lymph node is considered to be optional in the treatment of early-stage cervical cancer, while para-aortic lymphadenectomy is defined as mandatory in stages IB2 and IIA1. FIGO2021 and SEOM guidelines recommend Type C radical hysterectomy [30], which is always recommended in stages IB1–IB2 and IIA2 [21,28]. The type of surgical approach, whether MIS or open, is highly debated as it is influenced by the results of the largest randomized clinical trial LACC trial published in 2018, which would affirm the superiority of the open approach in the surgical treatment of early cervical cancer in terms of survival [4]. Similar results to the locally advanced cervical cancer (LACC) trial were found in the SUCCOR study published in 2020 [31]. The study indicated that in stage IB1 patients undergoing radical hysterectomy, the MIS approach, compared to the open approach, increased the risk of recurrence and death. This result was consistent even when avoiding the use of the uterine manipulator and implementing preventive maneuvers to minimize potential tumor spread during colpotomy [31]. In the ESGO2023 guidelines, MIS may be considered only in low-risk tumors (<2 cm and free margins after conization) and in high-volume centers experienced in performing MIS radical hysterectomy which meet the ESGO quality criteria for surgery, if the patient agrees [11]. According to JSGO guidelines, MIS is permissible for tumors with a diameter of 2 cm or less, provided that tumor spread is adequately prevented. However, it is discouraged for tumors larger than 4 cm. The decision is subject to appropriate counseling based on local and international evidence [15]. The FIGO report 2021 defines any surgical approach (abdominal, vaginal, or MIS) as being possible in stages IA1, IA2, and IB1. The AIOM 2022 guidelines contain a moderate recommendation, stating that laparotomy should be the approach of choice [17]. Recently, the SHAPE trial, a randomized clinical trial that compared radical versus simple hysterectomy in the surgical treatment of cervical cancer ≤ 2 cm, discovered that the simple hysterectomy is comparable to the radical in terms of the three-year incidence of pelvic recurrence [6]. The validation of these findings has recently been confirmed on two meta-analyses [32,33].

### 3.4. Sentinel Lymph Node Biopsy: State of the Art

In recent years, the role of the sentinel lymph node in gynecological oncology has become a prominent topic. It was incorporated into the American NCCN guidelines in 2015. Since then, there has been a substantial increase in understanding of its potential role in staging early-stage cervical carcinoma [34]. The NCCN 2024 and ESGO 2023 were mainly compared to enunciate the latest developments in this area; its execution requires specialized centers in gynecological oncology. The algorithm can be utilized when no suspicious lymph nodes are observed either preoperatively or intraoperatively. It recommends performing side-specific lymphadenectomy in all instances of non-capture on the ipsilateral side [35]. Nevertheless, it is still presented as an alternative rather than a primary recommendation in various specific cases. NCCN 2024 defines it as being considered for stages T1a2, T1b1, Tb2, and T2a2 [12]. In the corresponding stages, ESGO 2023 recommends the performance of sentinel lymph node biopsy, specifically emphasizing the importance of confirming their negativity through radiological imaging [11]. It is also advised to evaluate lymph node status during surgery using a frozen section analysis [11]. BGCS2020′s recommendations emphasize the importance of appropriate training in centers with a high caseload to ensure the highest quality [19]. Lastly, JSGO contemplates SNL in stages IA2, IB, and IIA, provided there is a well-trained team and collaboration with an adequately trained pathologist. For SNL analyzed using the ultrastaging method, a sub-classification of lymph node involvement was introduced in the latest TNM version, based on the size of the lymph node metastasis: (1) single tumor cells (ITCs) < 0.2 mm, (2) micrometastases 0.2 to 2 mm, and (3) macrometastases > 2 mm. In line with FIGO staging, only micrometastases and macrometastases count as positive lymph node involvements, while the role of ITCs is still not clarified [36].

### 3.5. Fertility-Sparing Treatment

Fertility-sparing treatment (FST) can only be undertaken in gynecologic oncology centers with comprehensive experience in this type of surgery. Each woman who wishes to preserve fertility and who has a histologic diagnosis of squamous cervical carcinoma or HPV-related adenocarcinoma ≤ 2 cm in maximum size should be informed about the possibility of FST after multidisciplinary consultations (ESGO2023). The purpose of this consultation should be to inform the woman about the possibility of abandoning the conservative strategy if positive margins or lymph node involvement are found, and about the oncologic and obstetric risks related to this kind of surgery. FST includes the following: conization, simple trachelectomy, and radical trachelectomy (performed abdominally, vaginally, or minimally invasively). ESGO2023 asserts that the negative pelvic lymph nodes’ (PLN) status is the precondition for any FST. Therefore, pelvic node staging should always be the first step of the procedure. For this aim, ESGO guidelines provide the SLN execution [9]; NCCN either pelvic lymphadenectomy or SLN; BGCS, FIGO, and SEOM consider only pelvic lymphadenectomy; and ESMO defines it as standard pelvic lymphadenectomy, but it affirms that SLN could be achieved. If intraoperatively proven PLN involvement is present, FST should be abandoned, and patients should be referred for CTRT and brachytherapy (BT). ESGO recommends that the primary objective of FST should be the resection of invasive tumors with sufficient free margins, coupled with the preservation of the upper part of the cervix. Therefore, an intraoperative frozen section is viable for assessing the upper resection margin. For ESGO, T1a1, and T1a2, either the LVSI-/+ or T1b1 LVSI stages are candidates for conization and simple trachelectomy. Type B radical trachelectomy is feasible in T1b1 LVSI+ [30]. NCCN2024 supports LVSI+ conization (with negative margins) in stage IA1 in addition to the SLN mapping algorithm or pelvic lymphadenectomy. Patients with stage IA2, IB1, and select IB2 could be eligible for radical trachelectomy +/− laparoscopic pelvic lymphadenectomy with or without SLN mapping. Recent evidence, even for patients in IB2–IIA stages of squamous histotype and adenocarcinoma, indicates that cold knife conization is a viable conservative treatment after neoadjuvant chemotherapy (NACT), demonstrating favorable outcomes in both obstetrics and oncology safety [37]. The ASCO guidelines are similar to the NCCNs, except that they do not cover the exceptional cases of IB2 stages. The ESMO guidelines also include the experimental option of NACT + FSS for tumors > 2 cm in size. BGCSs conduct a comprehensive analysis of various procedures, considering their indications based on the disease stage and the performing center’s capabilities. They are recommended as acceptable conizations alone for low-risk patients in stages below IB1. In stage IA2 with LVSI, however, a pelvic LND should also be performed [19]. They state it could be offered in IB1 patients enrolled in clinical trials. The AIOM guidelines do not include FST, while the FIGO cancer report 2021 and the Spanish SEOM guidelines only mention the possibility of performing conization in stage IA1 and radical trachelectomy in IA2 and IB1. JSGO suggests FST in stages IA1 for patients with a strong desire to conceive, LVSI-, negative margins, and no residual lesions evidenced with endocervical curettage specimens. For stages IA2-IB1, it recommends radical trachelectomy with pelvic lymphadenectomy. The indication for cerclage is addressed by ESGO and BGCS guidelines, and is mentioned by NCCN.

### 3.6. Treatment in Locally Advanced Disease (IIB–IVA)

The current gold standard of treatment is platinum-derived chemotherapy and concomitant external radiation therapy followed by brachytherapy. Surgical treatment in advanced stages is, to date, a viable alternative only within research protocols. Specifically, in the field of brachytherapy for LACC, the EMBRACE-1 study showed encouraging data on the efficacy in the long-term follow-up of chemoradiation therapy followed by 3D MRI-guided brachytherapy both in terms of organ morbidity and stable control [38]. Equally promising are the therapeutic options of carbon ion radiotherapy and chemo-carbo ion rt in the same patient setting, and even more so in the cases showing initial rapid regression of the tumor [39]. According to the latest ESGO guidelines, however, radical surgery may be considered in low-risk IB3 and IIA2 patients after appropriate evaluation of prognostic factors (tumor diameter, LVSI, and stromal invasion). The procedure should include an intraoperative assessment of lymph node status. In the case of their involvement in both macro- and micrometastases, the attempt of radical surgery should be abandoned. If surgery were to be performed, a C2-type radical hysterectomy [30] and pelvic and para-aortic lymphadenectomy to the level of the inferior mesenteric artery should be performed. Counseling to the patient should be targeted, considering any prognostic factors and following the principle of not combining surgery and adjuvant RT [40]. The patient should be discussed with a multidisciplinary team, and the advantages and disadvantages of both treatment options (surgery vs. radiotherapy) should be counseled about the individual prognostic factors. Given the limited number of patients with T1b3 and T2a2 tumors (<10%), referral to highly specialized centers is recommended. According to ESGO states, neoadjuvant chemotherapy (NACT) followed by radical surgery should not be performed outside clinical trials. ESMO guidelines in stages IB2 to IIIB include surgery as a possible alternative to radiation therapy after neoadjuvant chemotherapy or pelvic exenteration as an alternative to exclusive chemoradiation therapy in FIGO IVA stages. According to the FIGO Cancer Report 2021, when radiotherapy resources are limited, NACT is used to downstage the tumor, allowing for the improved safety of surgery [28]. In the latest NCCN 2024 guidelines, there was a disagreement between members of panel writers regarding the completion of surgery following primary chemoradiation for stage IB3 or IIA2 tumors in patients whose extent of disease or uterine anatomy precludes adequate coverage with brachytherapy. While adjuvant hysterectomy after RT has been proven to enhance pelvic control, its impact on OS is inconclusive, and it is linked to increased morbidity. A recent Cochrane review investigated the potential benefits of adding hysterectomy to standard non-surgical treatments for patients with locally advanced cervical cancer [41]. The findings indicate insufficient data to establish a survival advantage associated with surgery [41,42]. ASCOs reserve the option of hysterectomy in stages IIB and IIIA in a basic setting when chemotherapy is not consistently available and in stages IIIB to IVA in the same setting as adjuvant after chemotherapy. According to the AIOM 2022 guidelines, in patients with IB2, IIA, and IIB cervical carcinoma, neoadjuvant treatment to radical surgery should not be considered as a first-line treatment approach. The 2020 British guidelines consider pelvic exenteration to be a surgical chance in selected cases with stage IVA disease, especially when symptom control is needed, such as for fistulas. The principle asserted is to not combine radical surgery with adjuvant radiotherapy. The 2019 SEOM recommendations define that, in special LACC settings where RT is not available, NACT before surgery may be indicated (level of evidence IIa; recommendation grade B).

### 3.7. Adjuvant Treatment in Early Stage Cervical Cancer

The need for RT adjuvant to surgery is closely related to the presence of risk factors revealed on definitive histologic examination. Adjuvant radiotherapy treatment is generally indicated in the presence of at least 2 of Sedlis’ criteria [43]. Adjuvant pelvic RT versus no other therapy was evaluated in the randomized trial (GOG 92) of 277 selected patients with stage IB cervical cancer with negative lymph nodes after hysterectomy and pelvic lymphadenectomy. It was associated with a 47% reduction in recurrence (an absolute reduction of 12.6%) with an acceptable morbidity and a grade 3 or 4 adverse event rate of 6% versus 2%. At 2 years, relapse-free rates were 88% for adjuvant RT versus 79% for the “no adjuvant treatment” group. After a prolonged follow-up period of 12 years, the updated analysis reaffirmed that adjuvant pelvic RT contributed to increased PFS, accompanied by improved OS [44]. Adjuvant chemoradiation therapy is indicated in the presence of even only one of the following risk factors (Peters Criteria) [45]:

- Microscopic parameter infiltration;

- Lymph node positivity (macro or micro metastasis, sentinel lymph node or lymphadenectomy);

- Positivity of surgical resection margins.

However, risk factors for adjuvant treatment’s indication after radical hysterectomy are not universally recognized as Sedlis criteria in Asiatic, American, and European guidelines. The latest ESGO/ESTRO/ESP2023, JSGO2022, ASCO2022, FIGO2021, and ESMO2017 do not refer to Sedlis criteria in defining the risk of recurrence for adjuvant RT treatment. However, they all consider the same risk factors. JSGO takes the following additional step: it classifies the indication for adjuvant therapy based on risk factors, delineating three distinct classifications (high, intermediate, low), well-schematized, and easy to access [15]. Patients categorized in the intermediate group, specifically those in stage IB, after a simple hysterectomy, are considered to be suitable candidates for either RT or chemo-radiotherapy [15]. At high risk are patients with positive resection margins, positive pelvic lymph nodes, and the presence of parametrial invasion. For them, chemo-radiotherapy is recommended. Although Sedlis’ criteria are not mentioned, they correspond to the risk factors of patients considered at intermediate risk of recurrence in the ESGO2023, FIGO2021, JSGO, and ESMO guidelines. In the high risk of recurrence category, they all include positive or close surgical margins, positive lymph nodes, or microscopic parametrial involvement. According to ASCO, instead, in stages IB2 and IIA2, RT ± concomitant low-dose platinum-based chemotherapy after hysterectomy is indicated in the presence of negative margins or inoperable tumors [13].

### 3.8. Follow-Up Post-Treatment

The primary goal of follow-up is the early detection of recurrence so that treatment can be implemented as early as possible to improve survival. To date, however, studies on the efficacy of follow-up are conflicting; some studies report improved survival for treatment of asymptomatic recurrence, while others have not shown such an advantage [46,47]. Therefore, there is no clear evidence on the modalities of follow-up; the currently shared attitude results from the consensus among experts and literature reviews. However, follow-up should also be an opportunity to educate and support the patient after treatments and propose rehabilitation aimed at preventing and reducing the psychosocial, physical, social, and existential consequences of the disease and its treatment. In addition, the follow-up visit allows for the evaluation of the long-term results of new treatment strategies and the quality control of the treatment. These aspects are particularly emphasized in the ESGO2023 and British guidelines. NCCN highlights the importance of pelvic organ screening in the follow-up of patients radio-treated at high doses for exclusive purposes. According to ESGO, follow-up schedules can be individualized by considering prognostic factors, treatment modalities, estimated risk, and/or the occurrence of side effects. Follow-up intervals of 3–4 months for the first 2 years and 6–12 months up to 5 years are recommended. The NCCN, ESMO, AIOM, and BGCS also agree to this follow-up interval. In contrast, the SEOMs, distinguishing between low- and high-risk patients, recommend a 6-month follow-up for the first 2 years, while high-risk patients should be followed up every 3 months. According to ESMO and ESGO, imaging should be recommended only if clinically indicated. ESGO indicates MRI or CT scan as the first instrumental approach, while PET-CT if imaging is doubtful. The NCCN distinguishes the use of imaging in follow-up according to the stage of disease. In cases of stage I undergoing nonfertility-sparing surgery, imaging is indicated based on symptomatology and clinic suggestive of recurrence, except in patients with stage IB3 or patients requiring adjuvant treatment in whom PET-CT is recommended at 3–6 months after the completion of adjuvant treatment. In patients with stage I disease undergoing fertility-sparing surgery, pelvic MRI with a contrast medium is recommended at 6 months after surgery and then annually for 2–3 years. In patients with stage II–IV disease, NCCN recommends PET-CT (preferred) or chest–abdomen CT with contrast medium within 3–6 months after treatment completion or pelvic MRI with contrast medium 3–6 months after treatment. The most appropriate imaging method should be chosen for a patient with stage IVB or recurrence to determine the response or subsequent therapies. In cases of clinical suspicion of recurrence/metastasis, PET-CT and pelvic MRI are recommended for local evaluation. Cytology sampling is recommended annually by AIOM (weak recommendation) in all patients at least 6 months after completion of radiotherapy treatment. For NCCN, it is suggested, for example, in patients treated using FS surgery. While acknowledging the limited detection rate of recurrences using this approach, it emphasizes the significance of clinical assessment by experienced healthcare providers [10]. In women undergoing fertility-sparing treatment, HPV testing at 6–12–24 months with colposcopy (ESGO2023) is indicated. ESGO distinguishes follow-up procedures for women who have undergone exclusive chemo-radiotherapy. Given the challenge of objectively assessing certain aspects, imaging plays a more significant role in this context. It underscores that the instrumental examination for assessing the response to therapy should be the same as that used in the initial assessment and should be conducted no earlier than 3 months after the end of the treatment. Pelvic MRI is the best diagnostic tool to assess the disease’s local extension, while thoracic–abdomen CT or PET-CT is used to evaluate distant metastasis (ESGO23) [48]. Cytological sampling is not recommended in these patients. Especially in this group of patients, the concept of vaginal and sexual health education is reiterated; in cases of vaginal stenosis and dryness, vaginal dilatation with vaginal lubricants and local estrogen should be offered [49]. This indication is common in all guidelines. Without clinical indications, hematological tests or markers are not recommended (NCCN, ESGO, AIOM, BGCS, JSGO). CA 125 might find a rationale in the follow-up of cervical adenocarcinoma, as some data from the literature show that elevation precedes the diagnosis of abdominal recurrence [50]. For squamous cell carcinoma, the SCC marker has been shown to precede the clinical diagnosis of recurrence in 46–92% of cases with an advance of 2–7.8 months. The markers NSE and chromogranin A might be useful for small-cell neuroendocrine tumors [51], but the rarity of these histotypes does not allow clear scientific evidence. Undergoing validation is the ESGO calculator for risk of cervical cancer recurrence by Fischerova and Cibula. It is a model applicable for patients with early-stage cervical cancer (T1a–T2b) after primary surgical treatment, analyzing tumor histotype, maximal tumor diameter, grading, number of positive nodes, and lymphovascular space invasion. It is helpful for physicians to plan an individual surveillance strategy based on prognostic factors. Table 4 summarizes the main follow-up indications of the different scientific societies: ASCO is omitted, as it does not cover this kind of recommendation.

### 3.9. Management of Recurrences

Cervical carcinoma, when diagnosed at an advanced stage, exhibits a high recurrence rate, with the majority of recurrences occurring within the initial 2 years following diagnosis [52]. The sites with higher incidence of disease recurrence are the following: locoregional (pelvis, vaginal dome) and extrapelvic, in sites far from the irradiation field (aortic and supraclavicular lymph nodes, lung). The incidence of recurrence is variable depending on the following risk factors: stage (from 10% for stage IB up to 42% and 74% for stages III and IVA), lymph node involvement, margin infiltration, and depth of infiltration of the stroma. In the management of these patients, a multidisciplinary team (gynecologic oncologists, radiation oncologists, radiologists, pathologists, oncologists, urologists, and plastic surgeons) that collegially considers the patient’s history, focusing on the site of recurrence, treatment performed in the first instance, disease-free interval, as well as the symptomatology associated with the recurrence, is essential. Patients should always be centralized in tertiary care facilities with expertise in the field. Treatment patients should also be enrolled in experimental clinical trials (ESGO, NCCN, BGCS). If it is decided for salvage surgery, the intent must be to obtain a null residual tumor. Pelvic exenteration was introduced with palliative intent in cases of advanced cancer associated with pain, fistulas, or infections in patients unresponsive to radiotherapy. Subsequently, a few scientific studies have gradually published data supporting its potential primary role. Depending on the dimensions, anterior, posterior, or total exenteration is feasible via either a supravaginal or translevator route [53]. Chiantera et al. published results related to 167 patients with cervical carcinoma treated with pelvic exenteration, of which 16% were primary cases. They reported achieving complete resection (R0) in 72% of cases, a 5-year overall survival of 41%, a rate of major complications at 21%, an overall morbidity of 58%, and a perioperative mortality rate of 3% [54]. However, the majority of pelvic exenterations are still performed as a secondary procedure in patients with recurrent or persistent cervical cancer after primary radio (chemo)therapeutic treatment. A limited number of patients undergo primary pelvic exenteration for tumors or urogenital or genito-intestinal fistulas associated with radio-chemotherapy treatment. Although pelvic exenteration is the most common surgical approach in patients with isolated central pelvic recurrence, radical hysterectomy or brachytherapy may be considered in carefully selected patients with small, centrally located lesions (<2 cm) (NCCN 2024). In distinguishing between a curative and palliative intent, the most important negative prognostic factors include pelvic lymph node involvement, disease fixation to the pelvic wall, and positive surgical margins on the operative specimen. In the case of palliative exenteration, awareness of the limited life expectancy must be balanced with the palliative intent of pelvic exenteration. A satisfactory recovery after the procedure can take 3 to 6 months, and patients with residual tumors may not survive long enough to derive a significant benefit from the intervention. Nevertheless, evidence is starting to emerge in favor of the possibility of conservative surgery with the removal of only the site of disease recurrence versus exenteration. In 2021, considering that among the most frequent sites of central recurrence is the vagina, a retrospective monocentric study compared survival rates of patients undergoing vaginectomy alone for recurrence localized to the vagina versus those undergoing pelvic exenteration. Surprisingly, the research group found the validity of vaginectomy as a salvage therapy with minimal impact on the quality of life. These findings will undoubtedly warrant further confirmation [55]. Broadly speaking, the various scientific societies address the topic of cervical cancer recurrence by distinguishing between central and wall pelvic recurrences. The guidelines uniformly recommend a histologic diagnosis when suspicion of recurrence arises, particularly in cases of local recurrence. However, there is variation in the strength of this recommendation. ESGOs suggest it if feasible, NCCNs mandate it, and SEOMs strongly recommend it. In a basic setting, where pelvic exenteration is not viable, ASCO would recommend the consideration of palliative care only. FIGO2021 guidelines indicate good prognostic criteria in case of relapse, as follows: an isolated central pelvic recurrence with no involvement of the pelvic sidewall, a long disease-free interval from previous therapy, and the largest diameter of the recurrent tumor less than 3 cm [56]. However, ESMO guidelines provide only general treatment guidance based on OS and PFS data related to disease recurrence, lacking clear directives for individual relapse cases. Moreover, some authors report the possibility of performing pelvic exenteration through a minimally invasive approach in selected cases [57,58,59,60]; further studies are needed to confirm these results and whether they will have a place in future guidelines. British suggestions include a comprehensive evaluation of the surgical rationale for Lateral Extended Endopelvic Resection (LEER) as salvage surgery with the potential for achieving an R0 status. In line with Spanish indications, pelvic exenterations for lateral pelvic recurrences are recommended only for cases not involving the sciatic nerve. However, some retrospective experiences highlight that a Lateral Extended Pelvic Resection (LEPR) could also be a promising surgical solution for selected cases with isolated recurrence involving sciatic nerve, bone, muscle, or iliac vessels [61,62,63], if operated in a referral center. The JSGO guidelines mention pelvic exenteration or hysterectomy in cases of central recurrence in the vaginal stump or cervix in the previous radiation field. However, there is no clear distinction of treatment by type of recurrence (central, lateral pelvic); but it remains according to the therapies performed with the first diagnosis. In Table 5, there is shown a comparison of the management of local recurrences proposed by different scientific societies. If the recurrence is distant, with metastases proven on imaging, the treatment of choice is chemotherapy.

### 3.10. Cervical Cancer in Pregnancy (CCIP)

Cervical cancer is the most frequently diagnosed gynecologic malignancy in pregnancy, along with breast cancer, melanoma, and lymphoma. The most recent guidelines, ESGO2023, provide for the possibility of preservation of pregnancy in early stages of disease (IA1–IB2 according to FIGO2018), discovered in pregnancy, subject to ascertaining the absence of lymph node localization in the disease. Due to the low incidence of CCIP, centralization in a few well-equipped facilities is a must (ESGO23). BGCS provides an in-depth discussion of Cervical Cancer In Pregnancy (CCIP), with the review conducted by Prof. Amant. In 2009, guidelines derived from expert panels of the French Morice group [23], dedicated entirely to the treatment of cervical cancer in pregnancy, were being published in International Journal of Gynecological Cancer. Even for pregnant patients, there is the possibility of performing SLN using indiocyanine green [64]. Equally to the subsets of non-pregnant patients, it has not yet become a standardized procedure [64]. Due to ethical considerations, scientific evidence is limited, but existing case reports thus far suggest the safety and feasibility of its application [65,66].

Depending on the tumor stage and gestational week of pregnancy, the following treatment options should be discussed with the patient, including the following risks and benefits of each approach: 

- Adapted surgery, including tumor removal: conization, or trachelectomy are alternatives, both with lymph node staging according to the stage of the disease with the intent to preserve pregnancy. 

- Radical surgery or exclusive chemo-radiotherapy as recommended at the stage of disease without preservation of pregnancy, with or without previous termination of pregnancy. 

- Delay of cancer treatment until fetal viability (if possible > 32 weeks gestation) and initiation of tumor-specific treatment immediately after delivery by cesarean section.

- Chemotherapy until fetal maturity and initiation of tumor-specific treatment immediately after completion of delivery by cesarean section. 

- Treatment after delivery should consider any previous chemotherapy given. 

Patients with stage I disease who delay treatment until fetal maturity may undergo cesarean section with concomitant radical hysterectomy and pelvic lymphadenectomy (NCCN 2024). In its latest guidelines in 2019, resulting from a consensus meeting with all the top experts in the field, the INCIP group established that women pregnant at 22 gestational weeks should be the cut-off for deciding the type of treatment (FST, vs. CHT vs. NACT + FST vs. delayed therapy vs. termination pregnancy) and eventually the possibility to postpone treatment after delivery after this time, based on the clinical stage of disease [64]. Any FST in pregnancy requires the exclusion of nodal involvement exactly as a patient with the same diagnosis, but who is not pregnant. In the presence of lymph node positivity, a termination of pregnancy is imposed as the only chance to preserve the mother’s health [66]. In patients with locally advanced stage or residual tumor after conization that cannot be completely excised (risk of premature rupture of membranes and/or cervical insufficiency), platinum-based chemotherapy may be considered at the earliest from 14 weeks of gestation. Spontaneous delivery seems to have a negative prognostic impact in patients with CCIP. Therefore, a cesarean section after 32 weeks gestation (if possible) is the recommended mode of delivery. At the time of or after the cesarean section, stage-appropriate definitive therapy should be carried out in a manner corresponding to that of non-pregnant women. These are type D recommendations, and are thus derived from case reports, expert opinions, and, at most, from cohort or case–control studies. The indication for completion of delivery by cesarean section is recommended by all guidelines except for JSGO.

## 4. Discussion

In recent years, we have seen great changes in the treatment of patients with cervical cancer. The literature is rich in new approaches and guidelines that have undergone major changes. As with endometrial cancer, we wanted to review and compare the most recent guidelines following these changes [22]. This approach compared the main existing recommendations to determine the necessary updates to align with the rapidly evolving scientific research [67].

The outcomes from comparing the guidelines highlighted similarities, differences, and contradictions. While the surgical approaches may be similar with slight nuances, some follow-up indications and diagnostic work-up significantly differed. Contradictions exist, such as claiming that TNM pathological staging is no longer utilized, yet scattered references are found in various recommendations. Regarding the diagnostic process, it was noticed that EUA is not consistently featured in all guidelines, and when it is, its indication is not uniform. In a retrospective analysis, the German Gynaecological Oncology Registry has statistically shown that, in locally advanced stages of cervical cancer, clinical assessment of the parametrium under general anesthesia, along with intraoperative visualization of magnetic resonance images performed by a gynecological oncologist, demonstrated greater accuracy in detecting parametrial tumor involvement compared to relying solely on MRIs [68]. This aspect is far from being of secondary importance; as widely acknowledged, the assessment of parametrial infiltration is a crucial prognostic factor in determining the patient’s treatment. Similarly, despite contrary evidence in the literature, it has been highlighted that pelvic ultrasound performed by an expert sonographer is still not regarded to be on par with MRI as a diagnostic staging method by various scientific societies [69]. Nonetheless, compared to MRIs, ultrasound examination provides a dynamic assessment, enabling the operator to evaluate the sliding of contiguous tissues against each other. Moreover, as the transvaginal or transrectal probe is in direct contact with the disease-involved tissues, it enables a more reliable assessment of the potential tumor extension into the vagina. This dynamic aspect is also crucial in clearly delineating the relationship between the tumor and the pelvic wall. Even if it is not universally accepted, the available data would at least suggest a broader recognition of ultrasound in scientific recommendations [27]. Concerning the role of the sentinel lymph node, there are different indications regarding surgical staging in early-stage invasive cancers. Starting with an important fact, the overall survival rate after sentinel lymph node biopsy alone is above 90%, and it does not differ from the stratified survival data for pelvic lymphadenectomy [67]. Currently, leading scientific authors do not provide a uniform recommendation for its implementation. ESGO2023 recommends sentinel lymph node biopsy in T1a1 LVSI-positive stages, and affirms it to be provided in evaluations with a frozen section for T1b1, T1b2, and T2a1. On the other hand, NCCN2024 regards it as an alternative to pelvic lymphadenectomy in stages IA2–IB1 and LVSI-negative.

Sedlis criteria for choosing adjuvant treatment after radical surgery are not commonly addressed in the guidelines because they are still being debated. This is probably due to Levinson’s study from 2022, which established the need for histotype-specific nomograms, considering the Sedlis criteria defined on only squamous cell carcinoma data [70].

Concerning fertility-sparing treatment, a non-homogeneity of treatment choices, and above all, an uncommon treatment, emerges. This issue certainly needs to be reviewed, considering the epidemiology of the pathology. Moving to the follow-up scheme, the authors observed a sufficient consensus regarding the periodic timing that could be suggested, highlighting the absence of unequivocal recommendations on how to conduct it. In particular, discordance emerged about the indication of vaginal cytology. The variation in this picture raises significant concerns due to its heterogeneity among different scientific societies. Only the Italian AIOM guidelines indicate that cytological testing should be performed annually. In all others, the choice is considered optional, and is to be considered according to the investigator’s choice. Further research is needed to establish whether it is beneficial or desirable. Regarding the timing of periodic follow-up examinations, the significant heterogeneity underscores the necessity to identify a common standard or minimum consensus. A change will probably come from the ESGO group’s first results from the clinical application of the newly devised Annual Risk Calculation Model [71]. In managing recurrence, there is a potential shift in the approach from primary radiotherapy plus surgery (exenteration) to primary surgery plus chemotherapy and exclusive radiotherapy. However, this change is not uniformly observed across all guidelines.

Regarding the role of surgery as a treatment at primary diagnosis, the veracity of the ontogenetic theory on the spread of cervical cancer by Hockel et al. [72] is being investigated. They found that the tumor propagation is determined by cancer fields anatomically defined by the mature tissue derivatives of the morphogenetic fields. With these premises, he developed a surgical technique of Total mesometrial resection (TMMR) with therapeutic pelvic +/− lumbar/aortic lymphadenectomy without adjuvant RT in stages IB–IIA [72,73]. Two ongoing trials are now registered in clinical trials on the validation of ontogenetic theory in gynecological cancers (NCT02986568 and NCT01819077). This is also a hot topic because it denotes an active and opposed scientific double strand. TMMR, despite these promising data, is, to date, non-standard and is not mentioned in the guidelines [74,75,76].

Lastly, we eagerly await ongoing molecular research poised to reveal potential targets for therapy or prevention. The analysis reveals a positive correlation between cervical cancer and its stages with HPV types (clades), HPV integration, and certain risk-factor habits across different races and regions cohorts of patients [77]. Moreover, spheroids and organoids are revolutionizing research, particularly in the realm of gynecological cancers. New therapeutic possibilities are anticipated, which in vitro experimentation will help us uncover in cervical cancer [78].

Summarizing what the narrative review brought out and stimulated for future research, considering the ongoing trials, the future may challenge the current principle of avoiding the association of surgery with radiotherapy. Afterward, the outcomes of the ten-year SENTIX trial, showcasing the safety of sentinel lymph node biopsy in early-stage patients with good DFS, OS, and recurrence rate compared to pelvic lymphadenectomy, will likely prompt a wide update to treatment recommendations. Similarly, the anticipated indications for surgical radicalization based on the SHAPE trial and its meta-analyses are eagerly awaited. New perspectives in work could then come from reviewing the role of adjuvant radiotherapy in locally advanced stages. Trials are ongoing; for now, they represent a feasible option for some clinical recommendations. Furthermore, circulating cell-free HPV DNA has been suggested to gauge the severity of the disease [79]. It might be incorporated into the existing Sedlis criteria to guide adjuvant therapy for intermediate-risk patients [79]. In this way, more references will be available in the guidelines. Finally, immunotherapy has taken giant steps in recurrent, metastatic, and persistent cervical cancer that will increasingly need to be applied in gynecological oncology world centers.

The narrative nature of the review limits the work, despite conducting a comprehensive search of the latest scientific guidelines on the topic. The comparison of these guidelines revealed interesting points for future updates. However, the difficulty in treating cervical cancer in medium- to low-income regions poses significant challenges, such as the inability to implement the guidelines and a lack of medical resources in these countries. Another issue that somewhat weakens the study is the variation in public health insurance, an inherent and understandable challenge when making international comparisons of medical practices.

## 5. Conclusions

This review of the most recently included international guidelines about managing cervical cancer patients has unveiled discrepancies and omissions that will gradually need to be aligned with scientific evidence. We have attempted to discuss possible reasons behind these differences, intending to serve as a useful tool for authors in updating their main recommendations.

## 6. Future Directions

The year 2023 concluded with many new developments in cervical cancer research, and inevitably this will lead to a revision of the guidelines of major scientific societies in the coming months. Proof of this was the New Year’s Eve celebrated with the release of the 2024 NCCN guidelines. Significant updates regarding systemic therapy were just featured with the introduction of immunotherapy. In fact, as of January 2024, following the data presented at the ASCO 2023 congress, the FDA approved pembrolizumab in combination with chemoradiation therapy for stages III–IVA FIGO 2018 cervical cancer. Also encouraging are the prospects for the application of artificial intelligence in cervical cancer [80]; the first and greatest evidence has emerged for early screening of the disease, never forgetting that before we treat it, we want to prevent it.

## Figures and Tables

**Table 1:** References of the guidelines enrolled for the review.

Continent	Societies	Author	Year
America	NCCN [12] ASCO [14]	Abu-Rustum N.R. Chuang LT	2024 2022
Asia	JSGO [15]	Seino M.	2022
Australia	RANZCOG [16]	Blomfield P [13]	2007
Europe	ESGO/ESTRO/ESP [11] AIOM [17] FIGO [18] BGSC [19] SEOM [20] ESMO [21] INCIP [22] SFOG [23] Poland [14]	Cibula D. Pignata S. Bhatla N. Reed N. de Juan A. Marth C. Amant. F. Morice P. Basta T.	2023 2022 2021 2020 2019 2017 2019 2009 2019

**Table 2:** Diagnostic work-up. EUA: examination under anesthesia; tb-CT: total body CT; LN: lymph-node; /: not mentioned.

	ESGO	NCCN	AIOM	FIGO	BGCS	SEOM	ESMO
**EUA**	/	/	Yes (operator choice).	/	If necessary.	Yes, if not proper pelvic exam.	Yes.
**Ultrasound**	If trained sonographer.	/	Yes.	Available resources	/	/	/
**MRI**	Mandatory.	If indicated.	Yes.	Available resources.	/	If nodal disease.	
**Conization**	If necessary.	If necessary.		Always after biopsy.	/	/	Yes.
**PET-CT or CT scans; other imaging**	Alternatively, if LN is suspicious.		Tb-CT or only chest CT; 2°choice PET.	Symptom-based imaging; hysteroscopy if barrel cervix extended to vagina.	Rectal or rectovaginal exams recommended; imaging if necessary.	PET-CT if nodal disease.	Chest X-ray and intravenous pyelogram.

**Table 3:** Comparison of non-fertility sparing management in early stage cervical cancer in different guidelines. Cone: conization; SH: simple hysterectomy; SLN: sentinel lymph-node; RT: radical hysterectomy; PLND: pelvic lymphadenectomy; PALND: para-aortic lymphadenectomy; BT: brachytherapy. The symbols (*, **, ***; #, ##, ###) inserted serve to recall the indication given in the box. Line IIA1 has been removed, as the clinical indications are the same as those described for earlier stages in the corresponding guidelines.

	ESGO	NCCN	ASCO	AIOM	FIGO	BGCS	SEOM	ESMO	JSGO
**IA1, LVSI-**	Cone, no LN staging.	Cone or SH.	Cone or SH (basic); cone or SH or B type RH + pelvic LN if positive margins (maximal).	Cone or SH.	Only cone.	Only cone.	Cone or SH.	SH, cone-only if negative margins on frozen section.	SH.
**IA1, LVSI+**	Cone +/− SLN.	Cone biopsy + SLN/PLDN. Type B RH (modified radical hysterectomy).	As NCCN (basic); RH type B + PLND +/− paraortic (may offer SLN or RT if patient not fit for surgery) (maximal).	RH type B + PLND + salpingo-oophorectomy **.	SH + PLDN.	PLND or SLNB.	Cone or SH + PLDN.	SH + PLDN.	SH or RH + PLDN.
**IA2**	Cone or SH +/− SLN if LVSI +/−.	Type B RH (modified radical hysterectomy) + PLDN (+/− SLN) *.	SH (basic); (maximal): see ASCO IA1 LVSI+.	**	B type RH + PLND.	Cone/SH/RH (achieve clear margins).	SH + PLND +/− PALDN.	RH/SH + PLND +/− PALND, SLN.	RH + PLDN.
**IB1**	If LN + in imaging, only RT. SLN + frozen section: RH to be defined + PLND + salpingo-oophorectomy ***.	*	SH (basic); B type RH + PLND (SLN) (maximal).	**	C type RH + PLND.	RH + PLND + salpingo-oophorectomy.	C type RH + PLND +/− PALND #.	RH + PLND +/− PALND, SLN ##.	RH or RT-CHT.
**IB2**	***	*	NACT + SH (basic); RT-CHT + adjuvant hysterectomy (only if residual disease) (maximal) ###.	C type RH for correlation between tumor size and paracervix rish invasion.	C type RH + PLND.	RT-CHT + BT.	#	##	RH or RT-CHT.

**Table 4:** Follow up Scheme. N.R.: not recommended; VG: gynecological visit; mo: months; y: years; -: not detailed; LR: low-risk patients; HR: high-risk patients.

	ESGO	NCCN	JSGO	AIOM	FIGO	BGCS	SEOM	ESMO
**Timing**	ESGO Calculator.	VG every 3–6 mo for 2 y; 6–12 mo from 3 to 5 y; then annually.	VG every 3–6 mo for 2 y; every 6–12 for 5 y.	VG every 3–6 mo for 2 y; every 6 mo in next 3 y.	VG every 3–4 mo for 2 y; every 6 mo from 3° to 5°y; then annually for life.	-	LR: every 6 mo for 2 y; HR: every 3 mo for 2 y, then every 6 mo from 3° to 5°y.	VG every 3.6 mo in 2 y; every 6–12 mo until 5°y.
**Citology**	N.R.	N.R.	Suggested as needed.	Annually.	-	-	Only in irradiated pt.	-
**Imaging**	If symptoms.	If symptoms.	Suggested as needed.	If clinical indications.	Involved high pelvic lymph nodes, may justify interval imaging.	Not routinely.	-	Not routinely.
**Exams**	If symptoms.	Semiannual CBCs, blood urea nitrogen (BUN), and serum creatinine determinations.	Suggested as needed.	If clinical indications.	N.R.	-	-	-
**FU in FSS**	HPV test (6–12–24 mo).	Annual cervical/vaginal cytology, MRI at 6 mo, then annual.	Contraception for 6 mo; PMA counseling.	-	-	-	-	-
**Other**	Histology if recurrence suspected.	-	HRT recommender.	In previous RT-CHT-treated, limited pelvic examination, imaging and blood tests (including CEA, CA 125, CA 19.9, AFP, etc.).	-	-	-	-

**Table 5:** Management of local recurrences. Image-guided adaptive brachytherapy (IGABT); intra-operative radiotherapy (IORT); CTRT: chemio-radiotherapy; PE: pelvic exenteration; BT: brachytherapy; pt: patients. EBRT: external beam radiation therapy; R: recurrence; TA: thermal ablation, S-RT: stereotactic RT; RA: radiofrequency ablation.

	ESGO	NCCN	ASCO	AIOM	FIGO	BGCS	SEOM	ESMO	JSGO
**Central pelvic relapse after surgery**	CTRT + IGABT.	Surgery or EBRT +/− CHT.	CHT-RT or RT +/− BT. (maximal setting).	CHT-RT + BT.	PE (if pelvic wall and extrapelvic nodes are negative).	CT-RT or PE.	CRT +/− IMRT, BT.	RT +/− BT.	RT or CT-RT if localized, single to few lesions.
**Pelvic sidewall relapse after surgery**	RT if patient naive; extended pelvic surgery (LEER).	EBRT and/or CHT; resection +/− IORT or CHT.	CHT, tumor-directed RT, and palliative care.	CHT-RT.	CT-RT or PE.	Non-repeat previous therapy principle.	CRT +/− IMRT, BT.	-	-
**Central pelvic or sidewall after RT**	PE if central; if lateral, surgery in high experienced centers. CHT in non-suitable pt PE + IORT (Central); in <2 cm lesions, RH or BT.	PE + IORT (Central); in <2 cm lesions, RH or BT.	PE if central (enhanced setting); CHT, tumor-directed RT for pelvic sidewall. In maximal setting, Prior RT plus central disease: PE ± IORT or RH or BT (latter two “in carefully selected patients with <2 cm lesions”).	RH or PE.	-	RH or PE +/− IORT; LEER.	PE; RH in <2 cm central lesions: in lateral R, PE if sciatic nerve not involved.	-	Palliative CHT for symptom control; PE or RH if in vaginal stump or uterine cervix.
**Oligometastatic recurrences**	EBRT +/− CHT; nodal resection/debulking + RT; TA; BT or S-RT.	Surgery +/− EBRT; TA or RA +/− EBRT; or EBRT +/− CT.	-	-	-	-	CRT or RT (EBRT or S-RT); local resection, RA, S-RT.	-	-

## Data Availability

No new data were created or analyzed in this study. Data sharing is not applicable to this article.
